# Oxidants and Redox Signaling: Perspectives in Cancer Therapy, Inflammation, and Plasma Medicine

**DOI:** 10.1155/2017/4020253

**Published:** 2017-10-29

**Authors:** Sander Bekeschus, Lars Bräutigam, Kristian Wende, Eva-Maria Hanschmann

**Affiliations:** ^1^ZIK plasmatis, Leibniz Institute for Plasma Science and Technology (INP Greifswald), Greifswald, Germany; ^2^Department of Medical Biochemistry and Biophysics, Karolinska Institutet, Stockholm, Sweden; ^3^Department for Neurology, Heinrich Heine University Düsseldorf, Düsseldorf, Germany

Redox signaling is a key player in the regulation of physiological processes. Current concepts in redox biology and plasma medicine emphasize the significance of redox dysregulation in inflammation and different pathologies including cancer. The identification and characterization of specific protein thiol switches and redox-regulated signaling pathways are two of the challenges in the fields. For example, tumor cells are often localized in a hypoxic environment, which leads to a unique redox signaling affecting metabolism, proliferation, metastasis, and apoptosis, as well as angiogenesis and the immune response. Deciphering and understanding the redox regulation of particular molecules and processes in a physiological and pathological context could allow the development of new therapeutic avenues. These strategies could comprise development of small molecules specifically targeting thiol switches and dysregulated redox signaling cascades, or localized generation of reactive oxygen species, for example, by cold physical plasma sources. This special issue is dedicated to oxidants and redox signaling.

One of the papers is a review article entitled “Redox Regulation of Inflammatory Processes Is Enzymatically Controlled.” I. Lorenzen et al. introduce redox active molecules. They summarize distinct functions and pathological implications of the enzymes that regulate their production and decay, such as nicotinamide adenine dinucleotide phosphate oxidases (NOX), nitric oxide synthases (NOS), superoxide dismutases (SOD), and thioredoxin (TRX) family proteins. Moreover, the authors describe regulatory thiol switches in nuclear factor kappa B (NF*κ*B), a disintegrin and metalloproteinase 17 (ADAM17), and high mobility group box 1 protein (HMGB1) as well as in pathways related to inflammatory signaling including the TLR cascades.

In the article “The Synthetic Lignan Secoisolariciresinol Diglucoside Prevents Asbestos-Induced NLRP3 Inflammasome Activation in Murine Macrophages,” R. A. Pietrofesa et al. analyze the potential use of LGM2605 in chemoprevention of asbestos-induced mesothelioma. LGM2605 was formerly shown to induce gene expression of nuclear factor (erythroid-derived 2)-like 2 (Nrf2)-regulated antioxidants and to reduce cellular levels of reactive species, induced by asbestos. Here, the authors show that LGM2605 significantly reduces asbestos-induced expression of the NLRP3 inflammasome, iNOS, and NF*κ*B, as well as the release of proinflammatory cytokines, levels of nitrates/nitrites, and NF*κ*B activation.

The work entitled “Cold Atmospheric Plasma Induces Apoptosis and Oxidative Stress Pathway Regulation in T-Lymphoblastoid Leukemia Cells” by E. Turrini et al. aimed to mechanistically analyze the impact of cold atmospheric plasma and the induction of reactive oxygen and nitrogen species (ROS/RNS) on apoptosis, DNA damage, and the consecutive upregulation of redox-related enzymes, such as, superoxide dismutase, catalase, and glutathione reductase, thereby connecting the short-lived plasma-generated species to central cellular redox signaling pathways.

In the article “Toxicity and Immunogenicity in Murine Melanoma following Exposure to Physical Plasma-Derived Oxidants,” S. Bekeschus et al. demonstrate anticancer effects of cold physical plasma on melanoma cells in vitro. Specifically, these cells are subject to plasma-mediated oxidation, cell death, and reduced motility of the remaining viable cells. This is accompanied by alterations in biomechanical properties, that is, an increased stiffness and differential regulation of zonula occludens 1 (ZO1) proteins in melanoma cells following plasma treatment. Importantly, plasma treatment increases expression of major histocompatibility complex class I molecules and calreticulin, two major proteins for recognition and phagocytosis of antigen-presenting cells necessary to mount an immune response.

In the paper “2-Deoxy-D-glucose Restore Glucocorticoid Sensitivity in Acute Lymphoblastic Leukemia via Modification of N-linked Glycosylation in an Oxygen Tension-Independent Manner” by Z. Leni et al., a possible way is described, how to address the issue of chemoresistance in childhood leukemia. By feeding a glucose analog to different acute lymphoblastic leukemia cell lines, the authors could show efficient killing of cancer cells that was accompanied by endoplasmic reticulum stress and induction of the unfolded protein responses. Both processes are critical in eliciting immunogenic cell death that is in principle capable of driving inflammation and antitumor immune responses.

The work of L. Hu et al. entitled “The Protective Roles of ROS-Mediated Mitophagy on ^125^I Seeds Radiation Induced Cell Death in HCT116 Cells” uncovers a critical role of phagocytosis of damaged mitochondria in irradiation-induced cancer cell death. By exposing human colon cancer cells to iodide-derived irradiation in vitro, they found an upregulation of intracellular ROS and targets (e.g., hypoxia-inducible factor *α*, HIF1*α*; BCL2/adenovirus E1B 19 kDa protein-interacting protein 3, BNIP3; NIP3-like protein X, NIX) involved in the induction of mitophagy, protecting cancer cells from cell death. The authors suggested mitophagic pathways to serve as possible drug targets in the future.

In the review article “Oxidative Stress Gene Expression Profile Correlates with Cancer Patient Poor Prognosis: Identification of Crucial Pathways Might Select Novel Therapeutic Approaches” by A. Leone et al., the authors discuss the double-face role of reactive oxygen species in cancer initiation, progression, and prognosis. Their analysis, which extends over 6 distinct tumor types, is based on cancer-type-specific oxidative stress gene profiles and data from the Cancer Genome Atlas database. Among the statistically significant genes associated with cancer initiation and progression, the authors found Forkhead box M1 (FoxM1) and thioredoxin reductase 1 (TrxR1) to be critical for the regulation of oxidative stress levels in all analyzed tumor types. Moreover, A. Leone et al. discuss how the identified signaling networks correlate to cancer stem cell signatures and provide by that knowledge on which redox pathways could be prioritized for the development of novel therapies.

